# Near-fatal subdural empyema complicating a rapidly progressive orbital cellulites

**DOI:** 10.4103/0974-2700.66540

**Published:** 2010

**Authors:** Nicholas Robert Johnston, John Joseph Ah-Chan, Hans Robert Stegehuis

**Affiliations:** 1 Department of Ophthalmology, Dunedin Hospital, Dunedin, NewZealand; 2 Department of Ophthalmology, Palmerston North Hospital, Palmerston North, NewZealand; 3 Department of ENT, Palmerston North Hospital, Palmerston North, NewZealand

Orbital infections are a group of potentially life-threatening infections. They are more common in younger children and are often caused by sinusitis. Intracranial complications from orbital infections or the acute sinusitis which is often the underlying cause are not uncommon and are life threatening. Presentation with proptosis, a red eye, reduced or painful eye movements and decreased vision indicate an orbital infection till proven otherwise. Issues relating to the to presentation and management of an advanced case of orbital cellulitis are presented here.

There is a need for a high index of suspicion and thorough systemic examination of a febrile patient.

Combined medical, otorhinolaryngologic, ophthalmologic and neurosurgical management may be required.

A 15-year-old male presented to a regional hospital emergency department with a 2-week history of headache, fever, malaise, diarrhea and vomiting. He denied neck stiffness, rash, photophobia or previous orbital trauma. On examination, he was febrile (39°C), with a pulse rate of 110/ min, blood pressure of 140/90 mmHg and O_2_ saturation of 100% in room air. Clinical examination was unremarkable besides mild erythema and slight swelling of the left upper lid. There was no rash, neck stiffness or focal neurological deficits. Blood cultures and a full blood count were taken (as was department policy, C-reactive protein and Erythrocyte sedimentation rate were not taken). He had a normal white cell count. His chest X-ray was clear and urinalysis was normal. He presented during a meningococcal epidemic and thus he was kept for observation in the emergency department by the afternoon emergency department staff, with a provisional diagnosis of “febrile query cause-? Viral illness”.

Twelve hours following initial presentation, he became increasingly agitated and the periorbital swelling had increased markedly. He was assessed by the night emergency department staff. He was febrile, he had reduced visual acuity of 6/12 (not previously documented), proptosis of the left eye and he also had reduced range of motion.

At this time, the only likely diagnosis was an orbital abscess although the differential diagnosis included a high-flow carotid-cavernous sinus fistula, rhinoorbital mucormycosis, cavernous sinus thrombosis, dacryoadenitis and idiopathic orbital inflammatory disease (orbital pseudotumor). He was given intravenous ceftriaxone and an urgent computed tomography (CT) scan of the orbits and sinuses was requested.

**Figure 1:**
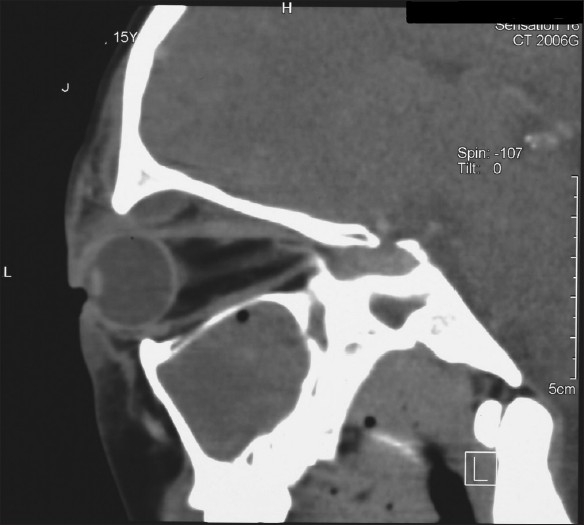
Sagittal computed tomography scan with contrast through the left orbit showing superior abscess and proptosis

**Figure 2:**
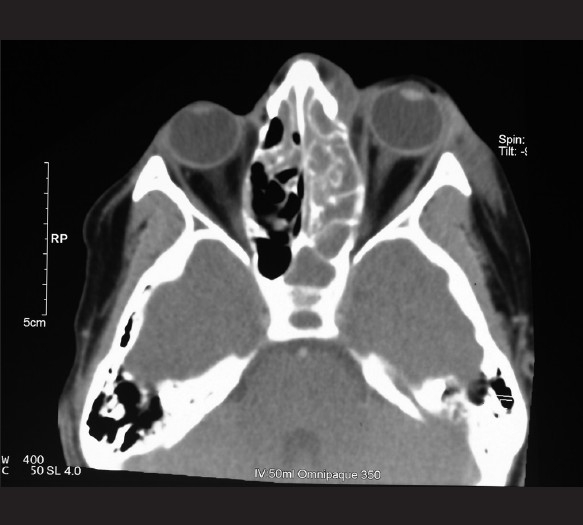
Axial computed tomography scan with contrast showing ethmoid sinus disease and proptosis

The CT scan showed gross bilateral paranasal sinusitis with opacification of the left frontal sinus and ethmoidal sinuses, and left-sided proptosis and a superolateral subperiosteal orbital abscess [Figures 1–2]. He was seen by otorhinolaryngology and ophthalmology and was taken to the theater for external drainage of the subperiosteal abscess as well as a left frontal sinus trephine- and bilateral maxillary sinus washouts.

The next day when he became agitated and confused the radiologist was questioned about whether the CT included the brain, and if so did it show any evidence of intracranial infection. The data available included most of the brain although not the occipital area. The radiologist was reasonably confident all she could see was normal with one minor reservation. An urgent magnetic resonance imaging of the brain was ordered. This showed a parafalciform subdural empyema with a left occipital cerebral abscess, an area of the brain not included in the CT. He was transferred to the neurosurgeons at a tertiary center where he had bilateral craniotomies to allow drainage of the subdural empyema and then functional endoscopic sinus surgery for his sinus disease. Blood cultures and sinus cultures grew *Streptococcus anginosus*.

The post-operative course was complicated by a right-sided hemiparesis, an abducens nerve palsy and seizures. Fortunately, following 6 weeks of rehabilitation, his only impairment on discharge was a higher-order balance deficit noted by the physiotherapist on functional gait and balance testing. He had normal visual acuity and normal visual fields and he did not have proptosis or limited ocular range of motion.

This case highlights the importance of considering the diagnosis of orbital cellulitis early. If his periorbital swelling had been recognised as a potential sign of orbital infection, he may have been investigated and referred earlier, allowing earlier surgical intervention. The severe complications of a subdural empyema and an occipital cerebral abscess with the accompanying craniotomy might have been avoided..

Lid swelling with proptosis, red eye and pain on eye movements, especially with restricted eye movements, are all signs of an orbital infection/inflammation. Reduced visual acuity with a relative afferent pupillary defect suggests optic nerve compromise.

A classification of orbital complications of sinusitis was described by Chandler[1] as: Group I, inflammatory edema (pre-septal cellulitis); Group II, orbital cellulitis; Group III, subperiosteal abscess; Group IV, orbital abscess; Group V, cavernous sinus thrombosis.

A subperiosteal orbital abscess (SPOA) is located between the periorbita (orbital periosteum) and the adjacent bone.[2–4] SPOA is most commonly medial due to ethmoid sinusitis. SPOA typically occurs due to ethmoid sinus disease due to the poor barrier to infection provided by the lamina papyracea.[2–4] Superolateral SPOA are normally related to frontal sinusitis and, usually a pansinusitis is present, as in our case. Frontal sinusitis is rare before the age of 7 years as the frontal sinuses have not usually formed till this age.[5,6] In a study of children between the ages of 3 and 14 who had CTs for rhinosinustis, only 7% of the 7–8-year-olds had frontal sinusitis compared with 15% of the 11–12-year-olds.[5] The mode of disease spread from the frontal sinus to the superior lateral SPOA is postulated to be via bacterial thrombophlebitis through valveless veins, through bony erosion or via congenital or acquired bony dehiscence.[3] There are three common sites for frontal sinus dehiscences, which may predispose a patient to superolateral SPOA. These include behind the trochlear fossa, behind the supraorbital notch and at the junction of the middle and outer thirds of the sinus floor.[3]

Medical management with intravenous antibiotics, covering *Staphylococcus aureus* and upper respiratory tract pathogens, is often effective as there is likely to be a single organism in the abscess.[2–4] The antibiotics should also have cerebral spinal fluid penetration as intracranial complications are common.

External drainage of a periorbital or orbital abscess is required if there isn’t a good response to the antibiotics, if the eye is at risk or if there is any evidence of an intracranial complication. Surgical treatment of the diseased sinuses, which may include frontal sinus trephination, should be done at the same time.[2,4]

Orbital infections are important to diagnose and treat early as they can lead to disabling or fatal consequences. CT scanning of the orbits and sinuses is a key investigation. Often, a combined medical, otorhinolaryngologic, ophthalmologic and neurosurgical management is required.
